# Ventral and dorsal streams processing visual motion perception (FDG-PET study)

**DOI:** 10.1186/1471-2202-13-81

**Published:** 2012-07-16

**Authors:** Sandra Becker-Bense, Hans-Georg Buchholz, Peter zu Eulenburg, Christoph Best, Peter Bartenstein, Matthias Schreckenberger, Marianne Dieterich

**Affiliations:** 1 Department of Neurolog, Ludwig-Maximilians University, Campus Grosshadern, Marchioninistr 15, Munich 81377, Germany; 2 Department of Nuclear Medicine, Johannes Gutenberg-University, Langenbeckstr 1, Mainz 55101, Germany; 3 Department of Neurology, Johannes Gutenberg-University, Langenbeckstr 1, Mainz 55101, Germany; 4 Department of Nuclear Medicine, Ludwig-Maximilians University, Campus Grosshadern, Marchioninistr 15, Munich 81377, Germany; 5 German Vertigo / Dizziness Center (IFB LMU), Ludwig-Maximilians University, Campus Grosshadern, Marchioninistr 15, Munich 81377, Germany

**Keywords:** Self-motion perception, Circular vection, Visual pathways, Ventral and dorsal stream, Visual-vestibular interaction, PET, Humans

## Abstract

**Background:**

Earlier functional imaging studies on visually induced self-motion perception (vection) disclosed a bilateral network of activations within primary and secondary visual cortex areas which was combined with signal decreases, i.e., deactivations, in multisensory vestibular cortex areas. This finding led to the concept of a reciprocal inhibitory interaction between the visual and vestibular systems. In order to define areas involved in special aspects of self-motion perception such as intensity and duration of the perceived circular vection (CV) or the amount of head tilt, correlation analyses of the regional cerebral glucose metabolism, rCGM (measured by fluorodeoxyglucose positron-emission tomography, FDG-PET) and these perceptual covariates were performed in 14 healthy volunteers. For analyses of the visual-vestibular interaction, the CV data were compared to a random dot motion stimulation condition (not inducing vection) and a control group at rest (no stimulation at all).

**Results:**

Group subtraction analyses showed that the visual-vestibular interaction was modified during CV, i.e., the activations within the cerebellar vermis and parieto-occipital areas were enhanced. The correlation analysis between the rCGM and the intensity of visually induced vection, experienced as body tilt, showed a relationship for areas of the multisensory vestibular cortical network (inferior parietal lobule bilaterally, anterior cingulate gyrus), the medial parieto-occipital cortex, the frontal eye fields and the cerebellar vermis. The “earlier” multisensory vestibular areas like the parieto-insular vestibular cortex and the superior temporal gyrus did not appear in the latter analysis. The duration of perceived vection after stimulus stop was positively correlated with rCGM in medial temporal lobe areas bilaterally, which included the (para-)hippocampus, known to be involved in various aspects of memory processing. The amount of head tilt was found to be positively correlated with the rCGM of bilateral basal ganglia regions responsible for the control of motor function of the head.

**Conclusions:**

Our data gave further insights into subfunctions within the complex cortical network involved in the processing of visual-vestibular interaction during CV. Specific areas of this cortical network could be attributed to the ventral stream (“what” pathway) responsible for the duration after stimulus stop and to the dorsal stream (“where/how” pathway) responsible for intensity aspects.

## Background

Under normal circumstances self-motion is perceived during motion of the head and body. However, apparent self-motion can also be elicited by large-field visual motion stimulation, during which the stationary subject misperceives the moving surroundings as being stable and himself as moving (i.e., vection) [1]. The direction of vection can be either linear, i.e., induced by optical flow stimulation in depth, or circular (CV), induced by observer-centered rotating visual stimuli. Newer studies showed that illusory self-motion can also be induced in the absence of an explicit motion signal, called “implied motion”, e.g., static elements of moving objects [2,3]. Vection is always accompanied by postural readjustments with head and body displacements in the direction of the visual stimulus [4-6].

Only a few functional imaging studies have investigated circular and linear vection [7-12]. Earlier water activation (H_2_^15^O)-PET studies on CV versus random dot movement or a stationary dot pattern showed a network of positive responses in parietal areas bilaterally. These areas were located specifically in a medial parieto-occipital area (PO) and the region of the intraparietal sulcus (IPS). Likewise there were extensive activations of the striate and extrastriate visual cortex including the motion-sensitive area MT/V5 and MST (BA19/37) in the temporo-occipital junction [8,10]. Simultaneously, signal decreases, i.e., deactivations, were found in multisensory vestibular cortex areas located primarily in the posterior insula and retroinsular regions (covering the parieto-insular vestibular cortex, PIVC) [8,10]. This activation-deactivation pattern led to the hypothesis of a tight inhibitory interaction between the visual and vestibular systems [8]. The same network of activations was also seen in fMRI by comparing phases of object motion with experienced self-motion in roll [9] or even with self-motion in depth [12]. Thus, apart from visual cortex areas some higher-order multimodal areas in the parieto-occipital cortex (IPS, PO) were involved in the processing of vection [13,14].

However, these older imaging studies were not designed to attribute specific aspects in the processing of circular or linear vection to a specific area within the network. Since the perception of vection requires that sensory aspects have to be integrated with motor processing, such as head and body tilt, we were interested in special subfunctions of this network. The first study dealing with specific functional differentiation used vestibular galvanic stimulation in fMRI [15]. The authors were able to attribute aspects of velocity of the vestibular stimulus (frequency) to certain components of the multisensory vestibular network, especially to those involved in vestibular processing at an earlier stage (e.g. posterior insula, posterolateral thalamus). Therefore, the goals of the present study were first, to analyze the pattern of visual-vestibular interaction (activation-deactivation pattern) during CV and second, to answer the question, whether it is also possible to attribute specific aspects of perceived CV to single areas of the visual or the vestibular systems or to a set of these multimodal cortical areas.

We chose the fluorodeoxyglucose positron-emission tomography (FDG-PET) set-up for the following reasons. We were interested in a strong and longer-lasting perception of CV that can be reliably scored by the subjects as to intensity aspects. Furthermore, FDG-PET allows robust measurements of the duration of perceived vection after stimulus stop and of the motor consequences (angle of head tilt). All these covariates of CV can be correlated with the regional cerebral glucose metabolism, rCGM, during CV. Eliciting such a robust CV requires a large-field coherent visual stimulation in an upright body position outside the scanner, which is not possible with an MRI technique. To allow visual stimulation outside the scanner with changing of the body position but without diminishing the quality of the PET data, a radiotracer with a relatively long half-life period was necessary, e.g. the radioactive isotope of fluor, 18 F. For comparison of visual motion stimulation with and without CV and for control of the perceptual parameters, the subjects underwent a second PET scan using random dot stimulation, which did not induce vection.

## Results

### Perceptual parameters

CV stimulation (condition A): Twelve of the 14 subjects perceived a body tilt during clockwise visual motion stimulation; seven ipsiversive, and five contraversive to the stimulus direction. These 12 subjects estimated the intensity of the perceived body tilt during the stimulation to be between 1 and 6 on a scale of 10 (mean 2.5 +/− 1.4). The head of all subjects was tilted ipsiversive to the stimulus direction, between 5° and 30° (mean 11.2° +/− 6.6°). Furthermore, all 14 subjects perceived a body tilt and vection once the stimulation stopped after 33 to 36 seconds (mean 34.8 +/− 1.6 s). The degree of perceived body tilt and head tilt, and duration of vection were distributed equally among subjects and gender. No subject reported vegetative sensations during the CV scanning period (A), although all subjects ranked the degree of unpleasantness between 1 and 7 on a scale of 10 (mean 3.0 +/− 1.5).

Random dot stimulation (condition B): This condition induced no apparent self-motion at all and was not perceived as unpleasant by eight of the subjects and as only mildly unpleasant by six of the subjects (between 1 and 2 on a scale of 10). No head tilts (0°) were measured. Therefore, correlation analyses could only be performed for the covariate of unpleasantness.

### Group categorical comparisons

#### CV vs. random

**Figure 1:**
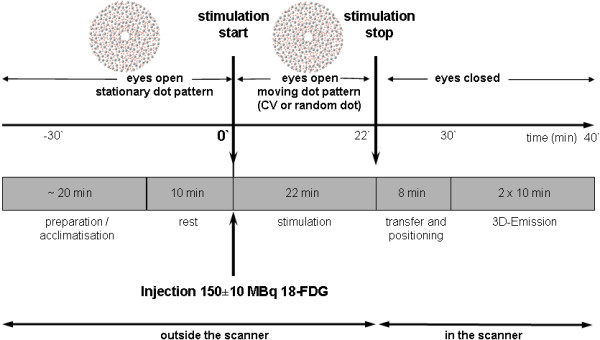
** Schematic drawing of FDG-PET imaging procedure.** After subjects were acclimatized by looking at the stationary dot pattern outside the scanner, the visual motion stimulation (CV or random dot movement) started simultaneously with the application of intravenous 18-FDG and continued for 22 minutes. Afterwards the subjects were quickly transferred to the PET scanner, where they immediately closed their eyes. The PET scans were measured under standard resting conditions in the same quiet and darkened room with eyes closed. The emissions scan started 8 minutes after the end of visual motion stimulation (30 minutes after injection) and continued for 20 minutes (standardized procedure).

The contrast between the rCGM during visual motion stimulation that induced circular vection and random dot stimulation without vection (contrast A vs. B) revealed cortical signal differences in the superior parietal lobule/precuneus bilaterally (BA 7; right: 263 voxels; left: 69 voxels), the right medial frontal gyrus (BA 8/9, frontal eye field), the right anterior cingulate gyrus (BA 24/31/32, t-value = 4.58), and in two parts of the right central region, most probably post-centrally (BA 3/5, 21/12 voxels) (Figure 1 bottom). The most significant signal differences were found in upper and lower midline structures of the cerebellum, e.g., the declive and folium (lobulus VI, VIIa) of the cerebellar vermis (partly tonsil and pyramid). The cerebellar activation of declive and folium was also evident at a threshold of p < 0.001 (t = 5.30, Figure 1, top and middle).

#### Random vs. CV

In the inverse contrast of random dot movement vs. CV only one small area in the left lingual and fusiform gyrus (BA 18/19, 111 voxels) was found.

### Group comparisons with age-matched controls at rest

To check the efficacy of both visual stimuli, their activation patterns were statistically compared with patterns of a second group of age-matched healthy controls, who had been scanned earlier in a complete resting state (no visual stimulation, eyes closed).

#### CV vs. rest

**Figure 2:**
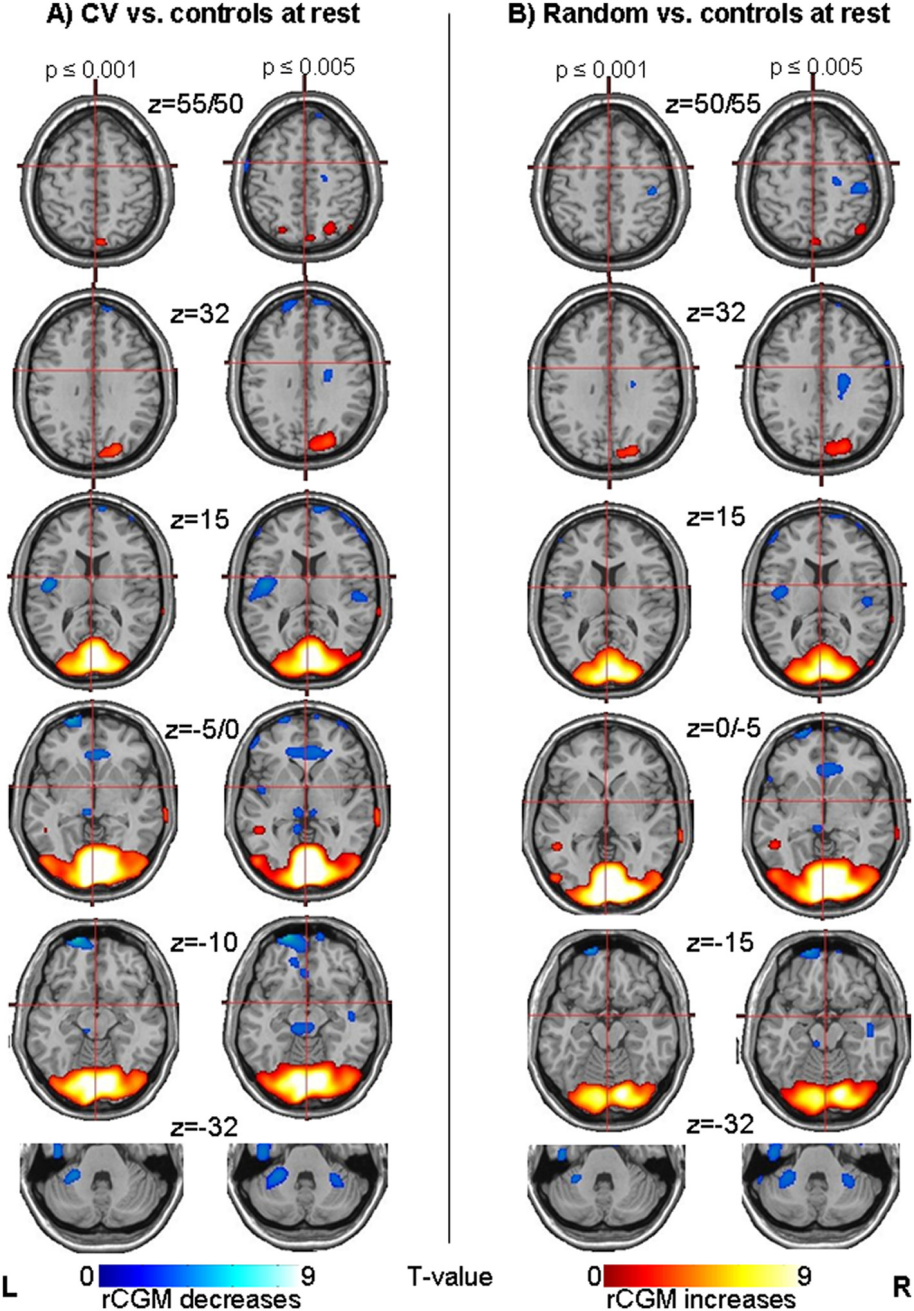
** Group categorical comparisons for circular vection (CV) vs. random dot stimulation.** Results of the group subtraction analysis of CV vs. random dot movement in 14 volunteers, showing the most significant activation in the midline structures of the cerebellum, i.e., the declive and folium of the cerebellar vermis, as well as parts of the tonsil and pyramid (upper and middle row). The activation cluster is projected onto a standard template provided by SPM in three different views (C = coronary, S = sagittal, T = transverse). At cortical level (lower row) signal differences were found in the superior parietal lobule/precuneus bilaterally (LPs/PCu, BA 7), the right medial frontal gyrus (GFm, BA 8/9, frontal eye field), the right anterior cingulate gyrus (GC, BA 24/31/32), and the right postcentral region (GPoC, BA 3).

This contrast revealed significant widespread differences in the visual cortex bilaterally (inferior and medial occipital gyrus, cuneus, lingual and fusiforn gyrus; BA 17–19; 16552 voxels) including the motion-sensitive area MT/V5 in the inferior/middle temporal gyrus (BA 19/37) and upper occipital areas (precuneus, middle occipital gyrus). Smaller separate clusters were found in the inferior parietal lobule/precuneus (BA 7/40) bilaterally and in the left middle temporal gyrus (BA 21/22) (Figure 2).

#### Random dot movement vs. rest

As expected, this contrast revealed an activation pattern similar to that of the contrast CV vs. controls at rest, including the visual cortex areas bilaterally (16038 voxels) as well as the right superior parietal lobule/precuneus (BA 7), and the left middle temporal gyrus (BA 21/22).

#### Rest vs. CV

The inverse contrast revealed signal differences due to rCGM decreases during the CV stimulation condition in parts of the vestibular cortical network such as the posterior insula bilaterally, and in the anterior cingulate gyrus (BA 32; Figure 2). Other signal decreases were located in the paramedian thalamus bilaterally, merging downward into the midbrain and upper pons, in the superior and medial frontal gyrus (BA 10/6), in parts of the lower superior temporal gyrus (BA 38) bilaterally, and in the middle cerebellar peduncle bilaterally, the left parahippocampal gyrus (BA 30/19), and the right precentral gyrus (BA 6).

#### Rest vs. random

The contrast showed a comparable pattern of signal decreases bilaterally in the posterior insula, anterior cingulate gyrus (BA 24/32), left pontine brainstem, as well as bilaterally in the fronto-temporal cortex, the middle cerebellar peduncles, and the central region predominantly in the precentral area (BA 6) at the border of the cingulate gyrus (Figure 2).

Thus, the patterns of both contrasts were confirmed to be similar for the two stimulation conditions (CV and random dot movement) as well as to patterns reported in earlier studies [7-10]. Consequently, the effects of the stimulations used gave a good basis for our correlation analyses.

### Correlation of rCGM and covariates of CV

**Figure 3:**
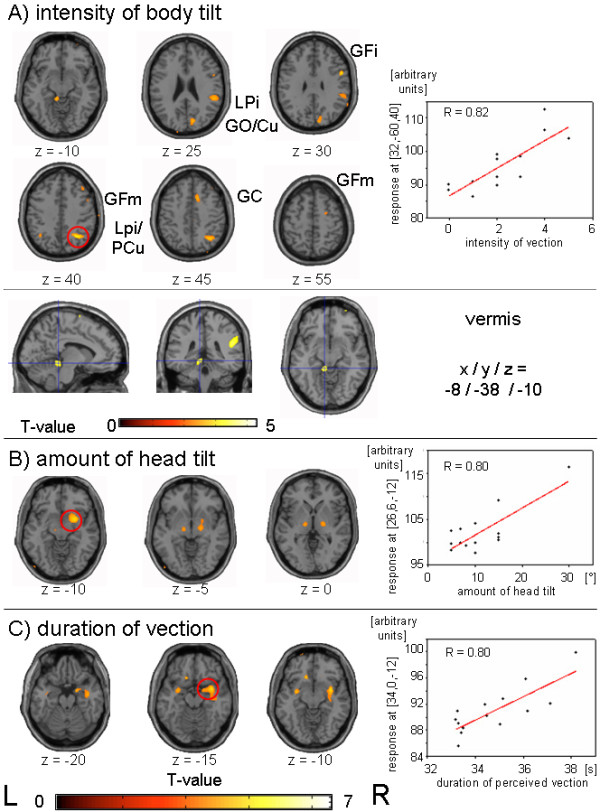
**Group comparisons with age-matched controls at rest (without stimulation, eyes closed)**. Results of the group subtraction analyses comparing the study data of CV, respectively, random dot movement stimulation, to an age-matched control group that was scanned earlier under identical conditions but with eyes closed and without any stimulation. For illustrative purposes activations (rCGM increases) and deactivations (rCGM decreases) are given at a threshold of p ≤ 0.001 and p ≤ 0.005.

For these statistical analyses the FDG uptake during the CV condition was correlated with each of the perceptual covariates for each subject. Figure 3 (A-C) shows all areas, in which the relative FDG uptake correlates significantly with the specific covariates of CV.

#### Intensity of perceived body tilt

Positive correlation was found for two areas at separate sites of the parietal cortex (BA 40/39; lower part of the right inferior parietal lobule/supramarginal gyrus, upper parts of the inferior parietal lobule/precuneus bilaterally) (Table 1; Figure 3: LPi, LPi/PCu). Other positively correlated areas were located in the right inferior frontal gyrus (BA 44/9; Figure 3: GFi), the medial aspect of the right frontal lobe bordering the anterior cingulate gyrus/limbic lobe (BA 32/6/8; Figure 3: GC), at two sites of the right medial frontal gyrus (BA 6 and 8; Figure 3: GFm twice), in the right upper occipital gyrus/cuneus (BA 18/19; Figure 3: GO/Cu) as well as in the culmen of the cerebellar vermis/parahippocampal gyrus left (Figure 3).

**Table 1:** Correlation analyses of rCGM and covariates of CV

**Intensity of body tilt**							
**Area**	**R/L**	**BA**	**X**	**Y**	**Z**	**T-value**	**Cluster**
*LPi/Cu*	*R*	*40/39*	*32*	*−60*	*40*	*4.88*	*323*
*LPi*	*R*	*40*	*52*	*−34*	*22*	*4.29*	*311*
LPi	L	40	−38	−58	40	3.58	42
GFm	R	8	42	32	42	3.38	50
GFm	R	6	26	−2	56	3.36	28
*GFi*	*R*	*44/9*	*54*	*6*	*32*	*4.52*	*111*
GFd/limbic lobe	R	6/8/32	12	14	44	4.12	117
GO/Cu	R	18/19	14	−78	24	3.98	168
Cerbellar vermis (Culmen)	L	-	−8	−38	−10	3.97	79
**Amount of head tilt**							
**Area**	**R/L**	**BA**	**X**	**Y**	**Z**	**T-value**	**Cluster**
*Thalamus/putamen/midbrain/subcallosal gyrus*	*R*	*34*	*26*	*6*	*−12*	*4.58*	*607*
Thalamus/midbrain	L	-	−14	−14	0	3.63	104
GFi/m	L	25	−12	20	−16	4.46	102
GTm/GTi	R	21/20	50	−2	−28	3.99	84
GF	L	18	−14	−90	−16	3.76	153
**Duration of perceived vection**							
**Area**	**R/L**	**BA**	**X**	**Y**	**Z**	**T-value**	**Cluster**
*GTs*	*R*	*38*	*34*	*0*	*−12*	*4.65*	*815*
GTs	L	38	−46	10	−30	4.11	475
*GTm*	*R*	*21*	*42*	*−4*	*−26*	*4.46*	*43*
GFm	L		−12	24	−12	4.40	58
GFm/s	R	10	24	40	14	3.41	15
Parahippocampal gyrus	L	-	−30	−2	−12	4.05	107
GOm	L	(19)	−30	−72	14	3.35	13

Areas showing positive correlations between rCGM and the different covariates of CV thresholded at p ≤ 0.005 uncorrected. For additional information, areas that are significant also at p ≤ 0.001 uncorrected are given in *italics*.

Abbreviations: Cu = cuneus; GF = fusiform gyrus, GFd = diagonal frontal gyrus, GFi = inferior frontal guys, GFm = middle frontal gyrus, GO = occipital gyrus, GOs = superior occipital gyrus, GTi = inferior temporal gyrus, GTm = middle temporal gyrus, GTs = superior temporal gyrus, LPi = inferior parietal lobule, LPs = superior parietal lobule.

#### Amount of head tilt

Positive correlation with the rCGM was mainly found for the lateral thalamus bilaterally, which merged on the right side into the adjacent parts of the putamen and subcallosal gyrus as well as bilaterally downwards in the midbrain (Figure 3). Other positively correlated areas were found in the left inferior/middle frontal gyrus (BA 25), right inferior/middle temporal gyrus (21/20), and the left fusiform gyrus (BA18) (Table 1).

#### Duration of perceived vection after stimulus stop

Positive correlation was found for areas in the right middle/bilateral superior temporal gyri (BA 21/38) and the adjacent parahippocampal and paralimbic areas (BA 21) (Figure 3). Smaller separate clusters were located in the middle/superior frontal gyrus bilaterally (BA 38/21/10), the left parahippocampal gyrus, and the left medial occipital/temporal gyrus (BA 19) (Table 1).

#### Unpleasantness

At the given threshold no area was found to be positively correlated with the degree of unpleasantness during the CV stimulation.

The correlation analyses also did not reveal significant results for the random dot condition.

None of the perceptual covariates showed a significant negative correlation with the relative FDG uptake. Thus, there were no correlations with areas with signal decreases.

## Discussion

The few earlier functional imaging studies during CV (circular vection – the visually induced perception of self-motion) revealed the presence of a cortical network within both hemispheres mainly located in striate and extrastriate visual cortical areas (V1, V2, V3a, MT/V5, MST) as well as in the medial parieto-occipital (PO) and intraparietal sulcus region (IPS) [7-10].

In the current study the categorical comparison for the contrast CV versus random dot movement yielded areas specifically related to CV. These areas of enhanced rCGM during CV included especially the upper parts of the cerebellar vermis (declive, folium, partly tonsil and pyramid), the superior parietal lobule/precuneus bilaterally (BA 40/7), the anterior cingulate gyrus, and the right post-central region (Figure 1) [8-12]. For new insights into the specialization of the different sites of this network for self-motion perception, we focused on correlation analyses of brain glucose metabolism in FDG-PET and different parameters of self-motion perception (perceptual covariates of CV). We were interested in special subfunctions of this network since perception of vection has to be integrated with motor processes related to head and body tilt. Furthermore, signal decreases have to be considered, because an older water activation PET study during CV reported that visual motion stimulation elicited a network of signal increases within the visual cortex that was accompanied by signal decreases, e.g., in the posterior insula, the parieto-insular vestibular cortex (PIVC) [8]. We, therefore, focused on areas involved in such visual-vestibular interactions.

### Visual-vestibular interactions during self-motion perception

The group subtraction analyses with age-matched controls revealed similar activation and deactivation patterns for both stimulation conditions (CV and random dot movement). In agreement with earlier studies [8-10], widespread activations were found in the visual cortex bilaterally (inferior and medial occipital gyrus, cuneus, lingual and fusiforn gyrus; motion-sensitive area MT/V5 in the inferior/middle temporal gyrus), upper occipital areas (precuneus, middle occipital gyrus), and smaller clusters in the inferior parietal lobule/precuneus bilaterally and the middle temporal gyrus. In the current study simultaneous signal decreases were located in parts of the vestibular cortical network such as the posterior insula bilaterally including the PIVC, the anterior cingulate gyrus, the thalamus and the superior temporal gyrus. This pattern had been interpreted to represent a reciprocal inhibitory interaction between the two sensory systems (the visual and the vestibular) during self-motion perception [8]. In contrast, two recent studies, one on coherent optical flow stimulation [16] and the other on egomotion stimulation induced by moving dots [17], found fMRI *activation* of the planum temporale/parietal operculum, probably also including the PIVC. This activation could be explained by the fact that the participants did not experience apparent vection. If there is no vection, there is probably also no relevant misleading visual-vestibular conflict that has to be reduced.

In line with this finding, another updated study on visually induced self-motion illusion in depth compared the illusion of self-motion to object-motion. The authors also did not find any significant deactivations associated with self-motion perception, but instead a similar activation network including the parietal, frontal, cingulate, and subcortical regions [12]. The authors speculated that the rotational self-motion reported in their current and earlier studies [8-10] and translational self-motion are processed differentially in the vestibular cortex. This interpretation is partly supported by an earlier PET study by Deutschländer and co-workers [10] in which roll vection caused a stronger deactivation of the area in the posterior insula than linear vection did. However, linear vection showed neither deactivation nor activation in the posterior insula [10].

The results of the current study give additional insights into the signal decreases: Whereas direct comparison of both stimulation conditions (CV vs. random) in the current study showed stronger activations of the precuneus and superior parietal lobule bilaterally, the anterior cingulate gyrus, and the cerebellar vermis during CV, there was no activation of the PIVC region. The inverse contrast (random vs. CV) showed no voxel at all, especially not in the region of the PIVC, where it was expected. Moreover, both stimulation conditions, random dot stimulation as well as the CV stimulation, compared to the controls at rest, resulted in a similar deactivation pattern including the PIVC in the posterior insula bilaterally (Figure 2). This is especially important, since it suggests that the deactivation of the vestibular cortex in the posterior insula (PIVC) is probably not directly related to a specific effect of the vestibular system during CV, but to motion stimulation in principle. Thus, the earlier hypothesis that the special condition of CV is probably encoded by the combination of simultaneous activations of parieto-occipital visual areas and concurrent deactivations of the posterior insula [8] has to be modified. On the basis of our current data, it seems more likely that CV is represented by a neuronal assembly of cerebellar vermal areas (involved in vestibular ocular motor processing) and specialized secondary visual areas such as precuneus/inferior parietal lobule/PO in association with deactivations of the “early” multisensory vestibular areas (i.e., PIVC). This interpretation is supported by recent animal data: A study found robust responses to 3D rotation and translation in the macaque retroinsular cortices, but no response of PIVC neurons to optical flow stimulation induced by random-dot stimulation [18]. The authors concluded that it is unlikely that the PIVC plays a significant role in visual/vestibular integration for self-motion perception. On the other hand, another animal study stressed the role of second visual areas such as the MST for the visual-vestibular interaction process, suggesting that the dorsal part of MST is an early stage of sensory convergence involved in transforming optic flow information into a reference frame that facilitates integration with vestibular signals [19]. Thus, the PIVC of the multisensory vestibular cortical system in humans might give input into the network when there is no primary vestibular stimulus (encoded as a deactivation) but a stimulation of the visual system only. This might induce the feeling of self-motion (encoded as activation of secondary multisensory areas in the temporal and parietal lobes).

### Neuronal representation of specific parameters during self-motion perception

To deepen our knowledge of specific aspects of CV processing at different sites of the cortical network, we performed correlation analyses of cerebral glucose metabolism and several parameters of visually induced self-motion (covariates of CV). A positive correlation between the *“amount of head tilt”* and the rCGM was found for basal ganglia areas such as the thalamus and putamen bilaterally and their adjacent inferior temporal areas. These are known to be responsible for the control of motor function of the head. In fact, an older SPECT study on head-down tilt in healthy volunteers found a significant increase in cerebral blood flow in the basal ganglia and the cerebellum [20].

The correlation parameter *“intensity of perceived body tilt“*identified areas known from brain imaging studies to be an integral part of the cortical network processing both vestibular information, such as the inferior parietal lobule/precuneus bilaterally right more than left (BA 40/39), anterior cingulate gyrus, cerebellar vermis, as well as the related eye movements such as the frontal eye field in the middle frontal gyrus. An activation of some of the multisensory vestibular cortex areas is of particular interest, since only visual (no vestibular) stimulation was performed in the current study. A closer look, however, confirms that these areas are not the vestibular cortex areas reported earlier to represent the center of the network in the posterior insula. During the last 10 years the cortical network of multisensory vestibular areas in humans has been defined by means of functional imaging studies using caloric, galvanic, and vestibular evoked myogenic potential (VEMP) stimulation [15,21-29]. This network parallels those of similar areas defined earlier in neurophysiological and tracer animal studies in different species (e.g., [30-34]; for review [35,36]. The different cortical areas (areas 2v, 3aV, 6, retroinsular regions, cingulum, IPL) are all connected to one area in the posterior insula, the so-called parieto-insular vestibular cortex (PIVC). It is thus assumed to be a “core region” of the multisensory vestibular cortical network in monkeys [35,37-39].

The inferior parietal lobule as well as the anterior cingulate gyrus found in the current correlation analyses belong to this cortical network of multisensory vestibular areas. The PIVC itself was not found in our correlation analyses for CV, nor were the neighboring areas in the superior temporal gyus and in the retroinsular region. This can be explained by the fact that it was not a primarily vestibular stimulus but a visual stimulus that induced the CV misleadingly perceived as self-motion. Thus, “earlier” multisensory vestibular cortical areas in the temporo-parietal lobe were not involved in the processing of the intensity of this visual stimulation, but instead multisensory parietal integration areas that are located in the transition zone between the temporal vestibular input and the occipital visual input and are more closely associated with the visual system. Accordingly, these areas do not belong to the so-called primary sensory areas, such as the primary visual cortex (V1) or the vestibular core region PIVC and its adjacent retroinsular areas [35]. Thus, the inferior parietal lobule and anterior cingulate gyrus seem to represent associative cortex areas relevant for special aspects of the sensory stimuli in general (visual, vestibular, and somatosensory), such as intensity or duration.

Moreover, the correlation analysis for the parameter *“intensity of perceived body tilt”* identified a specific area in the right visual cortex (cuneus, BA 18/19), the Talairach coordinates of which correlate with the parieto-occipital region (PO) found in earlier group subtraction analyses of circular and linear vection [8-10,12]. This result underlines the specific role of the parieto-occipital cortex not only for the processing of perceived circular vection per se but also for intensity aspects of body orientation in space. Other authors [17] assumed that this region represents the visual area pV6, corresponding to the V6 of Pitzalis et al. [40,41], which is involved in the extraction of optical flow cues for egomotion processing. This explanation nicely fits with our stimulus condition.

The correlation of the frontal eye field area with multisensory vestibular cortical areas indicates that there is a tight connection between the cortical ocular motor system and the vestibular network during perceptual tasks (intensity of perceived body tilt). Indeed, visual psychophysical studies have shown that the amount of optokinetic nystagmus (OKN) correlated positively with self-motion perception [42]. Our stimulation condition (the subjects looked relaxedly into the center of a large circularly rotating visual field) elicited no permanent OKN. Moreover, rotatory OKN as a response to a rotating stimulus is not easily induced in humans; it has to be trained [43], which was not done in our study. The relation to perception and not to continuous eye movements in this condition is also reflected by the fact that the cortical eye fields were not found in the group subtraction analyses of CV vs. random dot stimulation as well as vs. age-matched controls at rest.

Areas coding the “*duration of CV after stimulus stop*” were located in the more ventral medial temporal lobe bilaterally, i.e., middle/superior temporal gyrus and the adjacent parahippocampal and paralimbic area. This seems convincing, since these structures are known to play a crucial role in coding various aspects of memory processing reviews [44,45]; one important aspect is timing. A recent electrophysiological study in monkeys found that the hippocampus provided incremental timing signals from the presentation of one item to the next, whereas the perirhinal cortex signaled the conjunction of items and their relative temporal order [46]. However, a limitation of this correlation parameter is the small range of CV duration after stimulus stop, which lasted only seconds (see Figure 3C) and probably reflected a parameter of motion aftereffects.

Whereas older studies stressed the non-spatial memory functions of the human hippocampus, recent studies in animals and humans have revealed that vestibular and visual function is directly related to spatial memory, navigation, and hippocampal size [47-49]. New anatomical, electrophysiological, and imaging data support the view that vestibular input is primarily processed in the anterior part of the hippocampal formation, whereas visual cues are primarily integrated in the posterior part [50]. The activation clusters in our current study using visual stimulation to induce a “vestibular” sensation were located in the more ventral (i.e., “vestibular”) part of the hippocampal/parahippocampal formation bilaterally. This activation localisation is closely analogous to those of an FDG-PET study on visually induced self-motion perception in linear direction/depth [10], whereas an older PET study on circular vection [8] found the activation more posterior, probably due to methodological limitations.

Activation of the medial temporal lobe or hippocampus was also found in a recent fMRI study during retrieval of self-motion, i.e., active walking as well as passive transport [51]. The authors concluded that the medial temporal lobe is specifically relevant for retrieval of self-motion information. Furthermore, the temporal lobe belongs to the ventral “perceptual” pathways (“ventral stream”), which mediate perception of the visual world from early visual areas for cognitive operations [52].

## Conclusions

Our data provide evidence that visual motion stimulation with or without vection always interacts within the vestibular cortical network, mainly by deactivating vestibular cortical areas in the posterior insula (PIVC). During CV there is a significantly enhanced activation of the cerebellar vermis and parieto-occipital regions. Furthermore, correlation analyses indicate that the processing of stimulus duration is attributed to the ventral stream (“what” pathway), which is responsible for conscious visual perception, visual recognition, and visual memory. The intensity of perceived vection is processed in parietal areas and can be attributed to the dorsal stream (“where or how” pathway), which is mediated by a route running from the primary visual area to the parietal lobe. The dorsal stream provides mainly spatial information necessary for the planning and programming of motor actions [53-55].

## Methods

### Subjects

Fourteen healthy volunteers (mean age 29.5 +/− 2.3 years; 7 women, 7 men) without any history or complaints of neurological or neuro-otological dysfunction participated in the study after giving their informed written consent (CV and random dot stimulation). They took no drugs known to act on visual, ocular motor, or vestibular functions. The laterality quotient according to the 10-item inventory of the Edinburgh test [56,57] was determined because of the possible effects due to an earlier disclosed right-hemispheric dominance in the vestibular system of right-handers [23]. The test revealed a strong right-handedness in all subjects (mean laterality index score +100). The study was in accordance with the Declaration of Helsinki and was approved by the local Ethics Committee and the radiation protection authorities (BfS).

A second age-matched control group consisting of 14 healthy subjects (mean age 31.0 +/− 6.2 years), who had been scanned earlier under identical conditions without preceding visual stimulation (complete resting state) was used for an additional group subtraction analysis. This additional statistical verification was useful, since only two FDG-PET scans were approved for each of the subjects. It allowed us to check the activation patterns induced by both stimulus conditions per se (efficiency of CV and random dot movement) and to compare them with those in earlier studies on visual motion stimulation [8-10] in order to ensure that the stimulus was adequate.

### Visual stimulation and evaluation of vestibular correlation parameters

Self-motion perception was visually induced by a computer-animated dot pattern projected onto a large screen (2 x 3 m) that (A) coherently moved clockwise with a rotation velocity of the whole visual scene of 13.3 full rotations per minute or (B) moved randomly with the same speed and identical illuminance. Condition B did not induce any apparent self-motion, while condition A induced a strong feeling of being tilted to the subject’s right side. Simple moving dots were used as stimuli to induce motion, since they give no semantic information and do not activate higher-level brain areas as expected, e.g., in implied motion [58,59].

Due to methodological limitations (only two PET scans possible per subject) the moving stimulation for CV was restricted to the clockwise direction only, since in an earlier study [8] no relevant direction-specific difference of cortical activation pattern was seen for clockwise vs. counterclockwise stimulation direction. For visual stimulation the subjects sat outside the scanner in the PET room in a reclining chair in front of a large-field screen (2 m height x 3 m width) at a distance of 1.5 m and were asked to look in a relaxed manner at the dot pattern (without a central fixation target) throughout the entire stimulation periods. In this setup the visual field was completely covered (90° angle in horizontal and 60° angle in vertical direction), i.e., large-field stimulation. We chose the FDG-PET imaging technique and set-up, because it allows large-field stimulation outside the scanner and induces a much more robust self-motion perception for a longer time. During the 22 minutes of stimulus presentation the determining proportion of tracer accumulation in the brain is fulfilled.

For statistical correlation analysis three vestibular perceptual parameters were registered during the stimulation period. First, the *amount of head tilt* was measured 10 times during the stimulation period by a protractor (degrees of deviation from the plumb line) that was placed on the back of the subject. For further analyses the mean of these 10 measurements was used. Second, the subjects were interviewed after the stimulus exposure to estimate the *intensity of vection* experienced as body tilt during the stimulation on a scale ranging from 0 to 10 (from none to maximum) and to determine its direction (ipsi- or contraversive to the clockwise stimulus direction). As a third parameter, the *duration of perceived vection* after stimulus stop (in seconds) was determined; the subjects indicated this stop by signaling with a finger. The subjects were transferred to the scanner after the perception of vection ended. These latter vestibular parameters (covariates of CV) were chosen, because they could be easily obtained and reflect the perception of motion of the individual subject. Fourth, to differentiate activations due to the unpleasantness of the stimulus conditions the subjects were asked to score the perceived degree of unpleasantness between 0 and 10 (none to maximum). The subjects were also interviewed about autonomic side effects of the stimulation. All perceptual parameters were also evaluated for the random dot stimulation.

### FDG-PET imaging technique and image analysis

**Figure 4:**
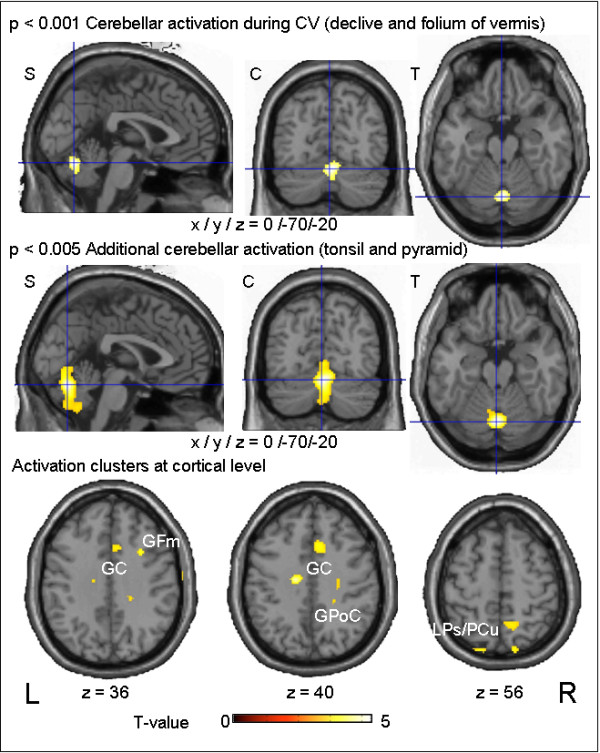
**Correlation analyses of rCGM and covariates of CV.** Positive correlations with rCGM for (A) the intensity of body tilt during CV, (B) amount of head tilt, and (C) duration of perceived vection after stimulus stop. For illustrative purposes the results are shown at a threshold of p ≤ 0.005. Scatter plots between the relative FDG-uptake and the different covariates of CV are given for the voxel with the maximum t-value (red circles indicate its location) as well as its correlation coefficients. Abbreviations: Cu = Cuneus, GC = cingulate gyrus, GFi = inferior frontal gyrus, GFm = middle frontal gyrus, GO = occipital gyrus, GOm = middle occipital gyrus, PCu = precuneus, LPi = inferior parietal lobule.

The healthy volunteers underwent two FDG-PET scans in an ECAT Exact PET Scanner (Siemens/CTI, Knoxville, TN) in random order over a 1-week interval; one scan was made after visual stimulation that induced circular vection (CV, condition A), and a second one after random dot visual motion stimulation that did not induce self-motion perception (condition B). Both stimuli were applied outside the scanner before scanning. The radiotracer was injected via an indwelling cannula that was placed in the vein of the left lower arm after an acclimatization period of 10 minutes while the subject looked at the stationary screens. The visual motion stimulation started simultaneously with the application of intravenous tracer (150 *±* 10 MBq 18-FDG) and continued for 22 minutes. Afterwards the subjects were quickly positioned in the scanner with the canthomeatal line parallel to the detector rings, to obtain transaxial images approximately parallel to the intercommissural line (AC-PC line). The transfer from the reclining chair to the scanner table took about 15 to 30 seconds; afterwards the subjects immediately closed their eyes. Both PET scans were measured under standard resting conditions [60] in the same quiet and darkened room with eyes closed. The emissions scan started 8 minutes after the end of visual motion stimulation (30 minutes after injection) and continued for 20 minutes in a three-dimensional acquisition mode (axial field of view 16.2 cm) (Figure 4). Attenuation correction was calculated using a computerized threshold limit routine (CTI software package) to define an isodensity contour of the maximum cerebral activity/pixel. The exact position of these isodensity contours was monitored visually slice-by-slice and was afterwards manually corrected. Forty-seven transversal slices, each 3.375 mm thick, were reconstructed using filtered back projection with a Hamming filter (filter width 4 mm). The scans had a transaxial resolution of 6.0 mm in the center of the field-of-view with full width at half maximum (FWHM) [61].

### Statistical analysis

Three-dimensional stereotactic surface projections (3D-SSPs) of the individual datasets as well as parametric z-score images were generated in a standardized manner in order to compare each individual subject’s data with a normal reference database consisting of 21 normal controls using NEUROSTAT (University of Michigan) as described earlier [60,62]. By this latter procedure, which is usually followed in clinical FDG-PET diagnostics, pathological cerebral glucose uptake patterns typical for neurodegenerative disorders such as Alzheimer’s disease or other central nervous system disorders could be excluded in the subjects.

PET data were further processed using statistical parametric mapping software (SPM2, Wellcome Department of Cognitive Neurology, London) http://www.fil.ion.ucl.ac.uk/spm. The PET images were realigned, spatially normalized into the standard anatomical space [63] defined by an FDG template according to Gispert et al. [64], and smoothed with a three-dimensional isotropic Gaussian filter using a 12 mm full width half maximum (FWHM) kernel. After proportional scaling of all PET scans to a mean global cerebral activity [65], t-statistical parametric maps (SPM [t]) were generated on a voxel-by-voxel basis using the general linear model [66]. Categorical comparisons of the two different stimulation conditions were performed by a paired *t*-test (CV vs. random; random vs. CV), and statistical correlation analyses were made between FDG uptake during the CV condition or during random dot stimulation, and the different perceptual covariates. For these correlation analyses the individual parameters belonging to each PET image were entered as covariates into the design matrix.

In a second analysis, the glucose metabolism of our two stimulation conditions (CV and random dot movement) was compared to that of the age-matched control group in a complete resting state by group subtraction analyses.

Regional cerebral glucose metabolism (rCGM) foci were considered significant for p ≤ 0.005 (whole brain, uncorrected) and if larger than ten voxels, according to the theory-driven a priori hypothesis for the visual [8-11] and vestibular areas [8,10,15,21-23,27,29] and based on the theory of random Gaussian fields [67]. For anatomical localization of clusters the MNI coordinates transformed to the Talairach Space using the mni2tal tool provided by CBU Imaging wiki ( http://imaging.mrc-cbu.cam.ak.uk) were used. The nomenclature of anatomical structures follows Talairach and Tournoux [68] as well as defined anatomical landmarks [69,70]. For identification of cerebellar structures the atlases of Duvernoy [71] and Schmahmann [72] were used.

## Competing interests

The authors declare that they have no competing interests.

## Authors’ contributions

SBB and MD conceived of the study, coordinated its course, and drafted the manuscript. HGB, MS and PB participated in the design of the study especially concerning the PET set-up and protocol as well as of the image analyses. HGB controlled the PET measurements and performed statistical analyses. PE programmed and realized the computer animated visual stimulation, and participated together with CB in the coordination of the study and measurements of perceptual parameters. All authors read and approved the final manuscript.
